# Epigenetic landscape of placental tissue in preeclampsia: a systematic review of DNA methylation profiles

**DOI:** 10.1186/s12884-025-07950-0

**Published:** 2025-09-29

**Authors:** Majid Zaki-Dizaji, Amir Ebrahimi, Reza Saeedinia, Zohreh Heidary

**Affiliations:** 1 Human Genetics Research Center, Baqiyatallah University of Medical Sciences, Tehran, Iran; 2 Department of Genetics, Tabriz University of Medical Sciences, Tabriz, Iran; 3 Student Research Committee, School of Medicine, Iran University of Medical Sciences, Tehran, Iran; 4 Vali-E-Asr Reproductive Health Research Center, Family Health Research Institute, Tehran University of Medical Sciences, Tehran, Iran

**Keywords:** Preeclampsia, Methylation, Placenta, EWAS, GWAS

## Abstract

Preeclampsia (PE) is a complex pregnancy disorder characterized by hypertension and organ dysfunction, significantly impacting maternal and fetal health. This systematic review investigates the epigenetic modifications, specifically DNA methylation patterns, in placental tissue from PE pregnancies. We conducted a comprehensive literature search across multiple databases including PubMed, Scopus, Web of Science, and Embase for studies published up to November 2024. The review analyzed genome-wide methylation studies and gene-specific methylation patterns in placental tissue, revealing significant differential methylation in numerous genes and pathways associated with PE pathogenesis. Our findings highlight distinct methylation profiles between early-onset and late-onset PE, tissue-specific methylation patterns within the placenta, and potential therapeutic and diagnostic implications. This review contributes to understanding the epigenetic mechanisms underlying PE and identifies promising biomarkers for early detection and intervention strategies.

## Introduction

Preeclampsia (PE) is a complex pregnancy-related disorder characterized by hypertension and organ dysfunction, typically arising after 20 weeks of gestation [1, 2]. It poses a significant risk to both maternal and fetal health, contributing to adverse outcomes such as preterm birth, fetal growth restriction, and maternal morbidity [1]. Despite advancements in our understanding of its clinical features and management, the exact etiology of PE remains elusive [3, 4], necessitating further exploration into the underlying biological mechanisms involved in its pathogenesis.

Recent studies suggest that epigenetic modifications, particularly DNA methylation, play a crucial role in the development of PE [5, 6]. DNA methylation, a covalent modification of DNA that involves the addition of methyl groups to cytosine residues, affects gene expression without altering the underlying DNA sequence [7, 8]. These modifications can be influenced by various factors, including maternal environment, nutrition, and other external stimuli, marking them as pivotal in gene regulation during critical periods of fetal development [9]. In placental tissue, which serves as the interface between mother and fetus, aberrant methylation patterns may disrupt normal placental function and contribute to the pathophysiology of PE [10, 11].

Given the complexity of PE, understanding its epigenetic landscape is essential for delineating the molecular pathways leading to its onset. A systematic review of the DNA methylation profiles in placental tissue from PE pregnancies could reveal significant insights into the disorder’s etiology and progression. This review aims to consolidate existing literature on genome-wide methylation changes in PE, identify key genes affected by these modifications, and explore their potential implications for both diagnosis and treatment. By providing a comprehensive overview of the epigenetic alterations associated with PE, we hope to pave the way for new avenues in research and clinical practice that could ultimately improve outcomes for affected individuals.

In conclusion, this systematic review seeks to encapsulate the intricate relationship between DNA methylation and PE in placental tissue, shedding light on how epigenetic mechanisms may influence this multifaceted disorder and contribute to its clinical manifestations.

## Methods

This systematic review was conducted according to the preferred reporting items for systematic reviews and meta-analyses (PRISMA) [12]. In addition, the study was carefully reviewed and received ethical approval from the Tehran University of Medical Sciences (approval code: IR.TUMS.IKHC.REC.1403.080).

### Search strategy

We conducted a comprehensive literature search to identify studies investigating DNA methylation profiles in placental tissue of PE pregnancies. The search included articles published up to November 2024 across multiple databases, including PubMed, Scopus, Web of Science, and Embase. Search terms combined MeSH terms and free text words related to"preeclampsia,""placenta,""DNA methylation,""epigenetics,"and their synonyms. 

### Inclusion and exclusion criteria

**Fig. 1:**
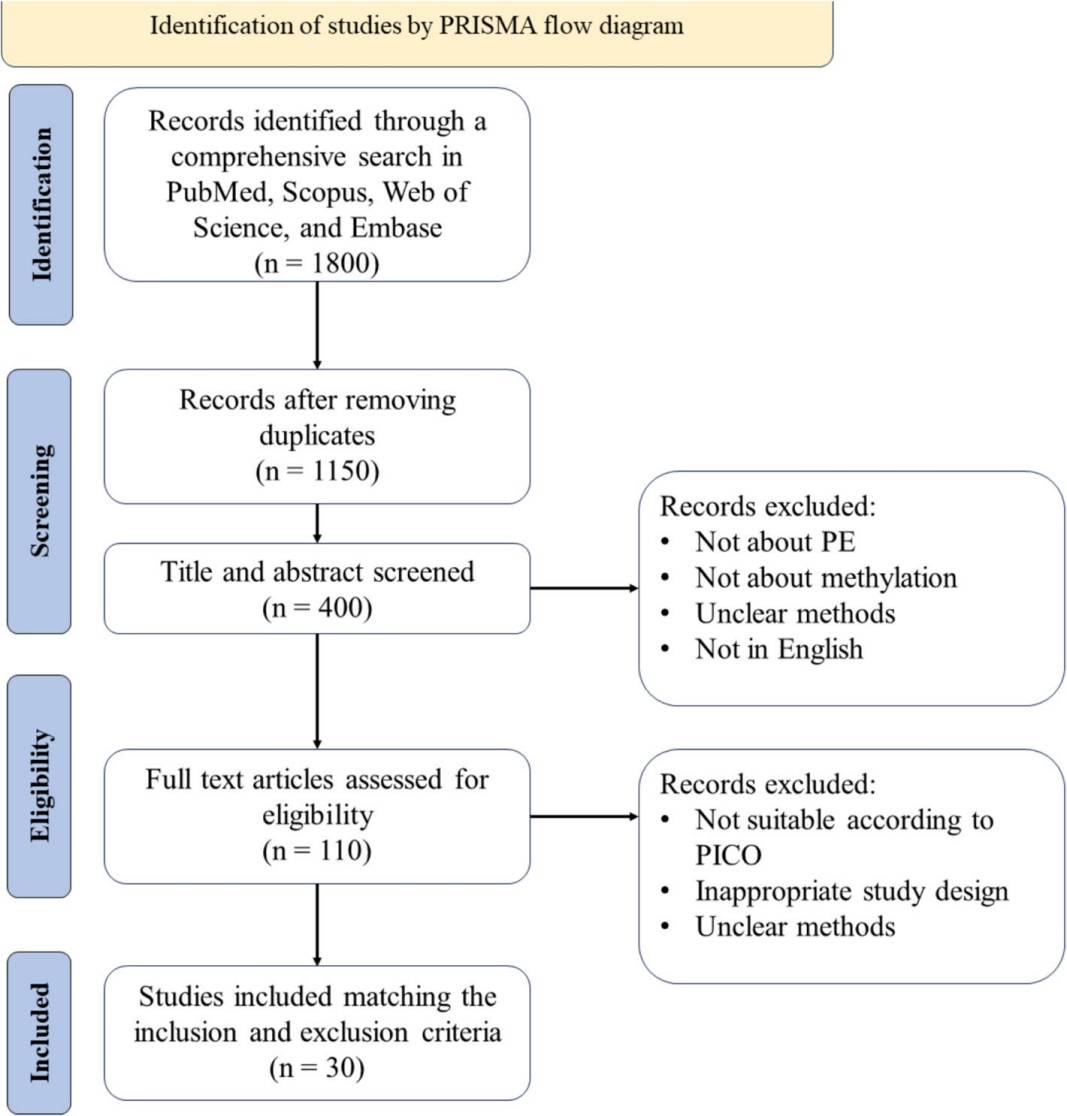
PRISMA flow diagram depicting the identification, screening, eligibility assessment, and inclusion process for studies in a systematic review. The diagram outlines the number of records at each stage, along with reasons for exclusion, ensuring transparency in the selection process

The inclusion criteria consisted of original research articles, studies focusing on DNA methylation in placental tissue, comparative analysis between PE and healthy pregnancies, published in peer-reviewed journals and the language should be in English. On the contrary, the exclusion criteria were articles such as reviews, meta-analyses, editorials, and conference abstracts, studies not involving human subjects, and articles that lack sufficient methodological detail for assessment (Fig. 1).

### Data extraction and quality assessment

Two independent reviewers (AA and MZ-D) screened the titles and abstracts of the identified studies. Full-text articles were retrieved for potentially relevant studies. Discrepancies were resolved through discussion or consultation with a third reviewer (ZH). Data extraction included: study characteristics (author, year, location), sample characteristics (sample size, population demographics), methodological details (DNA methylation analysis techniques, target genes or regions), and key findings (significant differentially methylated regions, biological relevance).

## Result

### Overview of included studies

**Fig. 2:**
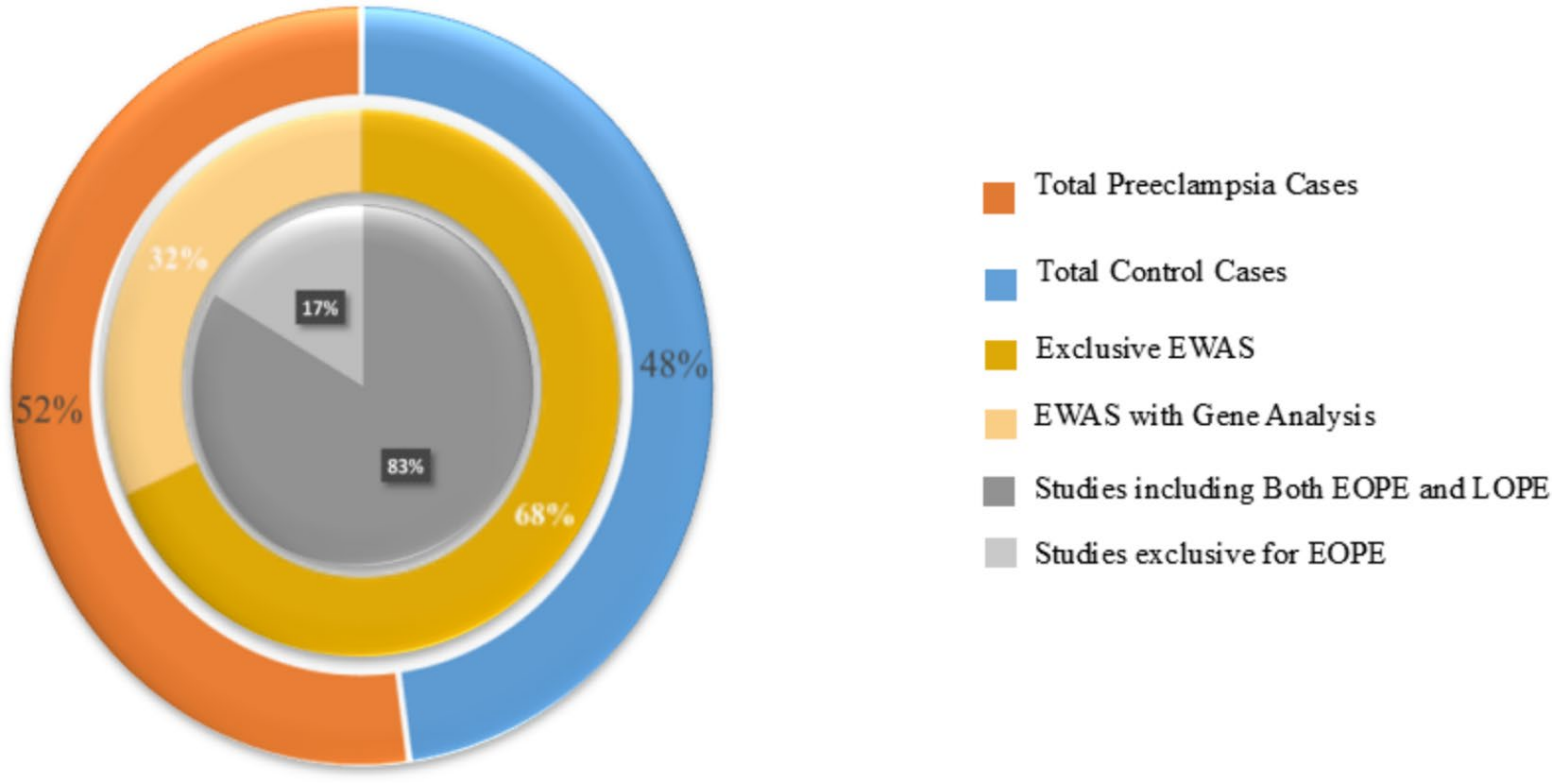
Circular summary chart illustrating the distribution of cases and study designs in preeclampsia research. The diagram categorizes preeclampsia and control cases, exclusive EWAS studies, gene analysis inclusion, and differentiation between EOPE and LOPE study cohorts, providing a comprehensive visual overview of the dataset composition

Based on our systematic review, a comprehensive analysis of the included studies reveals significant patterns in research methodologies and findings (Fig. 2). Among the 31 eligible articles identified, there was a total of 2270 participants, comprising 1086 PE cases and 1184 controls. The studies are from various countries (USA, China, Canada, Netherlands, Korea, Australia) spanning from 2010 to 2024, with sample sizes ranging from small exploratory cohorts to larger validation groups. Populations studied are classified based on clinical subtypes such as early-onset preeclampsia (EOPE), late-onset preeclampsia (LOPE), severe preeclampsia (sPE), term or preterm PE, and other related disorders (e.g., nIUGR).

Regarding methodological approaches, 19 studies employed epigenome-wide analysis (EWAS), with 13 using EWAS exclusively and 6 combining it with candidate gene analysis. The onset of PE was involved in 12 studies, with all these studies including cases of EOPE and LOPE, while only 2 included EOPE. Methodological quality assessment revealed that participant matching was majorly based on the gestational age.

This statistical overview highlights both the breadth of current research and areas requiring more rigorous methodological approaches in future studies. The predominance of cross-sectional designs and variable matching criteria suggests a need for more longitudinal studies with standardized methodological frameworks to better understand the temporal dynamics of DNA methylation in PE.

### Key findings on methylation profiles in placental tissue

#### Genome-wide DNA methylation changes in placenta

Genome-wide methylation studies in placenta tissue of PE patients are essential for understanding the disease's underlying mechanisms, identifying differentially methylated genes, and elucidating their roles in placental development and function. These studies contribute to our knowledge of how altered methylation patterns affect gene expression, which can lead to adverse pregnancy outcomes. They also offer insights into the influence of environmental factors on methylation changes, potentially leading to biomarkers for early diagnosis and risk assessment, as well as informing treatment and preventive strategies. ​Ultimately, such research enhances our understanding of epigenetic regulation in health and disease, with implications for developing targeted interventions in PE and other related conditions. According to the mentioned criteria, 19 distinct studies that has evaluated the genome-wide methylation patterns were recognized (Table 1). Within this research, 11 out of 19 studies utilized Illumina Infinium HumanMethylation450 BeadChip, and two used Illumina Infinium HumanMethylation27 BeadChip. The remaining six studies employed various platforms including: Illumina Infinium MethylationEPIC BeadChip v1.0 (commonly referred to as 850 K), NimbleGen Human DNA Methylation 3 × 720 K CpG Island Plus, NimbleGen Human CpG Island Plus Promoter Microarray (385 K), Illumina GoldenGate Methylation Cancer Panel I, and reduced representation bisulfite sequencing, with each platform being used once. Three studies validated their candidate genes by pyrosequencing, and one used MassARRAY EpiTYPER as an additional validation detection technique. Thirteen studies have narrowed down their results to identification of some candidate genes whereas the remaining six has reported the DMRs or DMGs without suggesting any candidate genes. Moreover, the affected underlying pathways and processes by these altered methylation patters were reported by ten studies. Ten studies have assessed the methylation patters in different stages of PE (early: EOPE and late onset: LOPE).

**Table 1:** Genome-wide DNA methylation

Author	Year	Country	Sample Size	Population	Method	Genes	DMR	Function	Ref
Leyman	2024	USA	EOPE^a^: 16 LOPE: 16 C: 10	EOPE LOPE	RRBS^b^	VEGFB PCDH9 PCDHA1-PCDHA13 LRP5 NKD1 KREMEN PRKCD	-	cadherin signaling pathway WNT signaling	[13]
Shuwei	2023	USA	Discovery: PE:28, C:28	-	Discovery: Infinium Methylation EPIC Bead chip	Tri 1: UBTD2 RAB18 CYP1A1	-	-	[14]
						Tri 2: UGP2 NCOA4 CHMP2A			
			Validation: PE: 64, C:50		Validation: Pyrosequencing	Tri 3: TRAF3IP2-AS1; TRAF3IP PSMA6 FOXK2			
Jiang	2022	China	PE: 20 C: 20	-	Illumina Human Methylation-27 Assay	PLXNB1 PMCH PPARG GOPC CD79A MME	-	-	[15]
Van den Berg	2020	Netherland	EOPE: 13 LOPE: 16 FGR: 27 PTB: 20 C: 36	-	Illumina HumanMethylation450K Bead Chip	BASP1 CRTAC1 IGF2BP3 NELL2 NRCAM RBP4 VGF	-	-	[16]
Lim	2020	Korea	sPE: 8 PE: 5 C: 13	-	Illumina Human Methylation 850 K Bead Chip	-	PE: 243 sPE: 155	Immune system disease Endocrine disturbance Seizure Thyroid disease	[17]
Leseva	2020	USA	Discovery PE: 19 C: 19	-	Discovery: Illumina Human Methylation 450 K Bead Chip	C12ORF10 MYG1 SDHAP3 ZNF300 SCUBE2 NAPRT1	-	-	[18]
			Validation PE; 16 C: 16		Validation: Pyrosequencing				
Wang	2019	China	PE: 22 C: 20	EOPE	Illumina Human Methylation 450 k Bead Chip MassARRAY EpiTYPER		2125 probes	trophoblast fusion	[19]
Wilson	2018	Canada	Discovery PE: 30 C: 43	EOPE LOPE	Illumina Human Methylation 450 k Bead Chip	CGA INHBA PAPPA2 ADAM12	Phase I EOPE: 1703 LOPE: 5	-	[20]
			Validation PE: 33 C: 15				Phase II: EOPE: 559		
Van den Berg	2017	Netherland		EOPE LOPE	Illumina Human Methylation 450 k Bead Chip	circadian clock and clock-controlled genes	6	internal body clock	[21]
Herzog	2017	Netherland	PE: 22 C: 60	EOPE LOPE UNCO FGR PTB	Illumina HumanMethylation450K Bead Chip	-	869	cardiovascular system	[22]
Yeung	2016	Australia	PE: 8 C: 16	-	Illumina HumanMethylation450K BeadChip Pyrosequencing	WNT2 SPESP1 NOX5 ALCAM	303	Wnt signaling Cell adhesion Fertilization and implantation Oxidative stress	[23]
Xuan	2016	China	PE: 6 C: 6	-	NimbleGen Human DNA Methylation 3 × 720 K CpG Island Plus	-	1664	-	[24]
Chu	2014	USA	PE: 24 C: 24	EOPE LOPE	Illumina HumanMethylation27 BeadChip	-	EOPE: 49 LOPE: 5	-	[25]
Ching	2014	USA	PE: 36 C: 36	EOPE	Illumina HumanMethylation450K BeadChip	GRB2 ATF3 NFKB2	9995 (900 genes)	cell cycle apoptosis cancer signaling inflammation	[26]
Anton	2014	USA	PE: 31 C: 14	Term PE Preterm PE	Illumina HumanMethylation450K BeadChip	CDH11 COL5A1 TNF NCAM1	229	Cell adhesion	[27]
Anderson	2014	USA	PE: 6 C: 6	-	Illumina HumanMethylation450K BeadChip	-	207	-	[28]
Blair	2013	Canada	PE: 20 C: 20	EOPE LOPE nIUGR	Illumina HumanMethylation450K BeadChip Pyrosequencing	INHBA BHLHE40 SLC2A1 ADAM12	38,840	Angiogenesis	[29]
Jia	2012	China	PE: 9 C: 9	-	NimbleGen human CpG island plus promoter microarray (385 K) BSP	CAPN2 EPHX2 ADORA2B SOX7 CXCL1 CDX1	296 DMGs	-	[30]
Yuen	2010	Canada	PE: 8 C: 9	EOPE LOPE	Illumina GoldenGate Methylation Cancer Panel I	TIMP3	EOPE: 34	-	[31]
Bourque	2010	Canada	PE: 17 C: 22	-	Illumina GoldenGate Methylation Cancer Panel I	H19/IGF2 ICR	-	Genomic Imprinting	[32]

^a^*EOPE* Early-onset PE, *LOPE* Late-onset PE, *FGR* Fetal growth restriction

^b^*RRBS* Reduced representation bisulfite sequencing, *BSP* Bisulfite sequencing PCR, *DMR* Differentially methylated regions

Recent analyses have demonstrated that distinct DNA methylation profiles in the placentas of PE patients are not only linked to the pathophysiology of the disorder but may also serve as valuable biomarkers for early detection. For example, Leyman et al. (2023) pinpointed differential methylation and expression of several genes (PLXNB1, PMCH, PPARG, GOPC, CD79A, and MME) in PE placentas, each with clear changes in expression [15]. A pioneering study, revealed significant differences in DNA methylation patterns, identifying 398 differentially methylated regions (DMRs), including 243 in PE and 155 in PE with severe features, when compared with normal placental tissues. Among these, 12 hypermethylated and three hypomethylated DMRs were found to be consistent across both PE groups, indicating their independent role from the severity of the condition [17]. Notably, the study highlighted the association of DMRs with various complications linked to PE, such as viral infections and immune system disorders, suggesting a critical role of epigenetic variation in modulating developmental processes and immune responses. To further substantiate the findings, researchers confirmed the hypermethylation patterns of DNA in HIST1H3E gene across both PE subgroups via quantitative real-time PCR. The results indicated a significant reduction in the placental levels of HIST1H3E in PE, affirming its hypermethylated status in both forms of PE when compared to controls.

Consistency in methylation patterns, such as hypomethylation of genes like TICAM2 and ZNF417, reinforces the significance of these loci in PE regardless of clinical severity [17]. Genome-wide approaches, including"bump-hunting,"have revealed hypomethylation in genes related to placental insufficiency and high uterine artery resistance, such as NAPRT1 [18].

Additionally, epigenome-wide association studies have uncovered thousands of hypomethylated probes in EOPE, including those linked to genes involved in trophoblast fusion [19]. Analyses of placentas from pregnancies featuring poor nutrient delivery have also displayed significant differential methylation, especially in EOPE cases [20]. Studies employing the Illumina Infinium Methylation 450 BeadChip have spotlighted alterations in cell adhesion and Wnt signaling pathways, while highlighting the broader impact of epigenetic disturbances on placental function and disease etiology [23]. Differential methylation has been observed between term and preterm PE, as well as between control and PE groups. Profiling promoter regions has revealed both hyper- and hypomethylated sites across various classes of CpG promoters, further expanding the scope of recognized epigenetic alterations in PE [27].

Additionally, another DNA methylation analysis demonstrated that of the promoters with altered methylation, a total of 1,664 were identified, which included 663 hypermethylated and 1,001 hypomethylated sites. Among these, high CpG–containing promoters accounted for 1,334, intermediate CpG–containing promoters for 383, and low CpG–containing promoters for 448 [24]. This extensive profiling indicates the expansive scope of epigenetic changes linked to PE and underscores the need for precise understanding of specific gene regulatory mechanisms.

Moreover, the research outlined the linkage between EOPE and distinct epigenetic alterations. A subsequent case–control study compared the genome-wide methylome differences in chorionic membranes from 30 EOPE and 17 full-term pregnancies, revealing a striking genome-wide hypermethylation pattern coupled with hypomethylation in promoters [26]. Out of 385,184 examined CpG sites, 9,995 demonstrated alterations, with 91.9% exhibiting hypermethylation. Importantly, this hypermethylation was closely associated with genes involved in critical pathways such as cancer signaling and inflammation, which may elucidate the biological functional alterations that occur in PE [26].

Further investigations explored distinct DNA methylation patterns in both maternal cells and fetal-derived tissue, providing insights into potential biomarkers for predicting PE and its inheritability in future generations. A prospective study evaluated genome-wide DNA methylation in first-trimester maternal peripheral blood and placental chorionic tissue from both normotensive and PE-affected women. This revealed 207 linked CpG sites with significant methylation differences, demonstrating the potential for early detection of PE through maternal epigenetic signatures [28].

Additional explorations focused on the implications of these epigenetic changes in maternal and fetal-derived tissues, identifying 207 CpG sites with significant methylation changes that could function as biomarkers for early detection of PE. This research revealed that these early epigenetic shifts might serve as indicators of heightened risk for LOPE in the mother [11]. Moreover, analyses targeting DNA methylation in chorionic villi samples from EOPE revealed a striking pattern of differential methylation that correlated inversely with gene expression, particularly in genes responsible for trophoblast function and angiogenesis [29]. Lastly, regarding the chromosomal locations of DMRs, Jia et al. identified 296 genes with aberrant methylation patterns mostly located in three chromosomes (1, 12, and 19) [30].

In conclusion, the exploration of DNA methylation in PE underscores its potential to illuminate underlying mechanisms, providing foundational insights into the disorder's pathophysiology. Such findings not only reveal critical epigenetic modifications associated with PE but also pave the way for potential diagnostic and therapeutic strategies aimed at addressing this complex and impactful disorder. Continued investigations into the epigenetic profiles of placentas in diverse populations will likely enhance our understanding of the interplay between genes and environmental factors in the etiology of PE.

#### Gene-specific methylation patterns in placenta

PE has been increasingly associated with oxidative stress and dysregulation of various genes related to methylation and expression in both placental and maternal tissues. This cumulative research emphasizes the intricate pathways involved in PE pathogenesis, exploring the roles of various genes such as SOD1, MMPs, TIMP3, CTGF, ALCAM, WNT2, and the vascularization genes VEGF, EGFR, and c-jun (Table 2).

**Table 2:** Gene-specific methylation dysregulations

Author	Year	Country	Sample Size	Population	Method	Gene	Region	Biologic impact	Ref
Deng	2024	China	PE: 20 C: 20	-	MS-PCR	11βHSD	Promoter	renin-angiotensin system	[33]
Zakeri	2023	Iran	PE: 20 C: 20	-	MS-PCR	SOD1	Promoter	Oxidative stress	[34]
Cruz	2022	Brazil	PE: 182 C: 147	EOPE LOPE Term PE	HumanMethylation450 BeadChip	TIMP3	Promoter	-	[35]
Zhang	2020	China	PE: 90 C: 94	-	MS-PCR	CTGF	Promoter	-	[36]
Wei	2020	China	PE: 47 C: 53	-	MS-PCR	ALCAM	Promoter	-	[37]
Boris	2020	Russia	PE: 26 C: 26	PE sPE	MS-HRM ^a^	TLR2 IGF2/H19 ICR	-	systemic inflammatory response	[38]
Zhang	2019	China	PE: 25 C: 50	PE PTB NTB	Pyrosequencing	DKK1 WNT2	Multiple CpG sites	-	[39]
Heil	2019	Netherland	PE: 22 C: 60	EOPE LOPE Control FGRPTB	Sequenom Epityper	LINE-1 PIGF	Multiple CpG sites	-	[40]
Rahat	2017	India	PE: 30 C: 90	-PE -Controls: First, second, trimester	MS-HRM	VEGF EGFR c-jun	Promoter	Vascularization	[41]
Liu	2017	China	PE: 16 C: 20	-	MS-PCR	WNT2	Promoter	Wnt signaling	[42]
Majchrzak-Celińska	2017	Poland	PE: 11 C: 25	-	MS-PCR Pyrosequencing	HSD11B2 RUNX3 LINE-1	-	-	[43]
Ge	2015	China	PE: 127 C: 132	-	MS-PCR	MTHFR	-	-	[44]
Tang	2015	China	PE: 20 C: 19	-	MS-PCR	HLA-G	Promoter	Immunity	[45]
Ma	2014	China	PE: 7 C: 22	-	MS-PCR	GATAD1	3’ region	syncytium deficiency	[46]
Zhuang	2014	China	?	-	COBRA MS- PCR DNA sequencing	Syncytin-1	Promoter	-	[47]
Xiang	2013	China	PE: 41 C: 22		MassArray EpiTyper	TIMP3	Promoter	-	[48]

^a^Methylation-Sensitive High-Resolution Melting curve analysis

Recent studies have focused on the relationship between oxidative stress and PE, noting the involvement of superoxide dismutase 1 (SOD1). Investigations have revealed significant changes in the methylation and expression of SOD1 in placental tissues, highlighting an increased promoter methylation rate (PMR) accompanied by a decrease in gene expression and enzyme activity, revealing that such epigenetic modifications are major contributors to the placental manifestations observed in PE [34]. Moreover, specific attention has been given to the DNA methylation (DNAm) profiles of matrix metalloproteinases (MMPs) and their tissue inhibitors (TIMPs), particularly TIMP3. Significant differential methylation of TIMP3 in placental samples from PE patients correlates with its altered expression, suggesting DNAm as a crucial regulatory mechanism in PE pathogenesis [35]. TIMP3 has emerged as a promising prenatal marker, especially in the Han Chinese population, where its hypomethylation and increased expression levels in PE indicate its relevance to the condition's etiology and offer potential for early diagnostic innovations in maternal plasma. This comprehensive exploration of the epigenetic landscape in PE not only elucidates potential pathways involved in its pathogenesis but also holds promise for developing early diagnostic tools and targeted interventions [48]. Similarly, EOPE is associated with hypomethylation of placental DNA, specifically lower levels of placental S-adenosylmethionine (SAM) and decreased DNA methylation of the PlGF gene. This suggests that placental DNA hypomethylation may play a role in the pathophysiology of EOPE, although further investigation is needed to confirm this relationship [40].

Connective tissue growth factor (CTGF) emerges as another important marker, with studies showing its upregulated expression correlating with decreased methylation of its promoter in PE placentas and maternal blood. Such findings underscore the potential involvement of CTGF hypomethylation in PE development, as lower methylation levels align with higher expression in trophoblast cells [36]. Additionally, research into the activated leukocyte cell adhesion molecule (ALCAM) has indicated that its gene promoter methylation is elevated in PE, opposing the downregulated expression, further emphasizing its probable role via methylation regulation in the disease's development [37].

Moreover, systemic analyses have identified that the methylation profiles of various genes, including IGF2/H19 and TLR2, fluctuate with PE severity. For instance, a decrease in IGF2/H19 ICR methylation is linked to a rise in systemic inflammatory responses in PE. These aberrations could be reliability detected in maternal plasma even before clinical manifestation, presenting these genes as significant early non-invasive markers for PE diagnosis [38]. Similarly, research into the methylation status of WNT2 and DKK1 suggests that disrupted methylation patterns in EOPE reflect changes in gene expression, illustrating their contribution to the molecular etiology of PE [39].

Studies also suggest that aberrant methylation plays a role in reducing placental S-adenosylmethionine levels in EOPE patients, implicating DNA hypomethylation as a pathophysiological factor in this condition [28]. Focused investigations into the methylation of vascularization genes such as VEGF and EGFR reveal alterations in their epigenetic landscapes in PE, pointing towards new diagnostic possibilities with VEGF promoter methylation patterns [41]. Similarly, aberrant WNT2 promoter hypermethylation correlates with diminished gene expression, calling for further research into its role in PE pathogenesis and potential treatment strategies [42].

Lastly, in the context of methylenetetrahydrofolate reductase (MTHFR), increased promoter methylation correlates with elevated plasma homocysteine levels in PE patients, indicating hypermethylation as a plausible causative factor in the disease [44].

#### Tissue-specific methylation within the placenta

Tissue-specific DNA methylation within the placenta is a fundamental mechanism regulating gene expression across distinct placental cell types, governing placental development, function, and adaptation during pregnancy. The placenta comprises a variety of specialized cells—including cytotrophoblasts, syncytiotrophoblasts, fibroblasts, endothelial cells, and immune cells such as Hofbauer cells—each exhibiting distinct DNA methylation signatures that underpin their functional specialization and contribute to the organ’s overall epigenetic landscape [49].

Recent cell-type resolved analyses have demonstrated that cytotrophoblasts, the progenitor cells within the placental villi, exhibit hundreds of differentially methylated CpG sites in autosomal regions compared with placental fibroblasts, underscoring the pronounced epigenetic heterogeneity between these populations [49]. For instance, cytotrophoblasts generally display lower methylation levels at certain regulatory gene promoters, which has been validated for genes such as the beta subunit of human chorionic gonadotropin 5 (CGB5), supporting cell-type-specific gene expression essential for hormone production and placental function [49]. Furthermore, these cell-type-specific methylation patterns are not limited to individual loci but can extend over broader regions, with cytotrophoblasts substantially shaping the overall placental methylome [50].

Spatial variation in DNA methylation is also observed across different regions of the placenta, correlating with both the spatial organization of cellular components and regional functional demands [51]. For example, differing methylation patterns are seen in the chorionic villi, anchoring villi, and basal plate, with implications for regional gene expression relevant to trophoblast invasion, angiogenesis, and placental barrier properties [52]. This spatial epigenetic architecture mirrors microenvironmental heterogeneity, such as regional variations in oxygenation and oxidative stress, which are themselves recognized contributors to PE pathogenesis [53].

In the context of PE, accumulating evidence points to the disruption of these refined cell-type- and region-specific methylation programs. Aberrant methylation specifically in placental cytotrophoblasts and in spatially defined regions has been linked to misregulation of genes involved in trophoblast differentiation, immune modulation, and vascular remodeling—processes central to the compromised placental function that characterizes PE [24]. For example, by using high-resolution techniques like the Illumina EPIC methylation array, researchers quantified methylation at over 850,000 CpG sites per cell type, revealing that each placental cell population possesses distinct methylation signatures. Trophoblasts showed the most unique methylation profiles, while Hofbauer cells displayed highly specific patterns that were markedly different even from other placental cells or blood-derived cell types [54]. This degree of cell specificity highlighted that previously observed “placental-specific” methylation marks vary considerably between cell types, emphasizing the crucial need to account for cellular heterogeneity in placental methylation studies [54]. The application of these cell-type specific methylation profiles has led to improved methods for correcting measurements of PE-associated differentially methylated cytosines (DMCs). In mixed placental tissue samples, methylation differences attributed to PE can be confounded by fluctuations in cell composition [54]. To address this, researchers used deconvolution techniques that leverage cell-type reference methylomes as a basis for estimating cell proportions in each sample. By integrating reference methylomes from the purified placental cell types, researchers can computationally correct for variable cell admixture, leading to more accurate identification of methylation changes truly attributable to disease processes such as PE, rather than to differences in cellular content [54].

The placenta serves as a pivotal mediator in maintaining a healthy fetal environment, accentuating its central role in PE pathophysiology and the influence of epigenetic programming on both fetal and placental development. A study embedded in the Rotterdam Periconceptional Cohort has underscored the potential link between altered circadian homeostasis and PE, revealing significant DNA methylation differences at multiple CpGs in both placental tissues and umbilical cord leukocytes (UCL) between EOPE, uncomplicated pregnancies, and GFR or spontaneous preterm birth cases. Notably, specific CpG sites demonstrated significant hypomethylation in placental tissue and hypermethylation in uncomplicated controls, implicating these epigenetic alterations in EOPE [21]. These findings emphasize the need for further investigation into tissue-specific variations and their broader implications on circadian performance and long-term health outcomes [21].

Epigenome-wide association studies further demonstrate that tissue-specific DMRs in the placenta are highly enriched for genes involved in embryogenesis, vascular development, and cell adhesion—biological pathways frequently implicated in the etiology of PE [22]. Importantly, the lack of comparable tDMRs in non-placental tissues, such as umbilical cord mononuclear cells, highlights the necessity of examining placental tissue directly when studying epigenetic mechanisms in PE [22].

Additionally, the disruption of the Catechol-O-methyltransferase (COMT) gene has been implicated in PE, with observed S-COMT (soluble COMT: the cytoplasmic, soluble form of the enzyme predominantly expressed in most tissues) promoter hypomethylation in placental tissues, contrasted by its dense methylation in blood samples, alongside no PE-specific methylation differences found between patient and control groups [55]. Despite this, the placenta-specific hypomethylation of the S-COMT promoter may serve as a potential early prediction marker for PE, necessitating further validation in maternal plasma screenings. This study emphasizes the intricate epigenetic landscape associated with PE while challenging a direct causal relationship between COMT promoter methylation and PE onset [55].

#### Impact of methylation aberrations on biological, cellular, and physiological processes with emphasis on gene-specific effects

The impact of methylation aberrations on biological, cellular, and physiological processes is profound, particularly when considering gene-specific effects in placental tissue. DNA methylation anomalies regulate gene expression in critical pathways involved in oxidative stress response, cell adhesion, trophoblast invasion, vascular development, and inflammatory modulation. For example, hypermethylation of the SOD1 promoter in PE leads to reduced gene expression and diminished antioxidant enzyme activity, thereby heightening oxidative stress within the placenta and contributing to disease pathology [34]. Similarly, hypomethylation of TIMP3 is associated with increased expression that affects extracellular matrix remodeling, positioning TIMP3 as a potential biomarker for early PE diagnosis [35].

In addition, decreased promoter methylation of CTGF results in its upregulation, which in turn contributes to fibrotic changes and an abnormal placental architecture characteristic of PE [36]. Elevated methylation at the ALCAM promoter suppresses its expression, undermining cell adhesion mechanisms that are essential for maintaining placental integrity [37]. Moreover, alterations in the methylation patterns of VEGF and EGFR disrupt angiogenesis and the proper development of placental vasculature, which can impair the supply of nutrients and oxygen necessary for fetal development [41].

Epigenetic modifications also extend to imprinted genes such as IGF2/H19 and TLR2, where changes in methylation status modulate fetal growth and inflammatory responses, offering deeper insights into the systemic epigenetic control over PE [38]. Disruption of methylation in key developmental signaling genes like WNT2 and DKK1 further interferes with trophoblast proliferation and invasion [39]. Increased promoter methylation of MTHFR is linked to higher plasma homocysteine levels, thereby connecting methylation status with metabolic risk factors in PE [44].

At the cellular level, these aberrant methylation patterns define the functional properties of specialized placental cells; for instance, hypomethylation at genes such as CGB5 is essential to ensure proper hormone production in cytotrophoblasts [49]. Physiologically, the resultant misregulation impacts the placenta's capacity for efficient nutrient and oxygen transport, alters immune tolerance, impairs vascular adaptation, and may even disrupt circadian rhythms, all of which converge to adversely affect fetal development and pregnancy outcomes.

In summary, these gene-specific methylation aberrations underscore the multi-scale impact of epigenetic changes on placental biology. They not only offer mechanistic insights into preeclampsia but also provide tangible opportunities for the development of biomarkers and targeted therapeutic interventions. A thorough understanding of this intricate regulatory network is fundamental to advancing maternal–fetal health in the context of preeclampsia and related disorders.

### Methylation changes in placental tissue across different stages of preeclampsia

In a previous mass spectrometry study conducted by our research group, 25 proteins were identified as differentially expressed in the cerebrospinal fluid of individuals with PE when compared to healthy controls. Building on this foundation, the current study aimed to investigate the DNA methylation of the genes encoding these proteins using an independent dataset derived from the Rotterdam Periconceptional Cohort. The study examined placental tissue, umbilical cord white blood cells (UC-WBC), and human umbilical vein endothelial cells (HUVEC) obtained from 13 patients with EOPE, 16 patients with LOPE, and 83 normotensive controls, which included patients with fetal growth restriction and uncomplicated pregnancies [16]. The analysis focused on 783 CpGs across 25 selected genes, where 15 CpGs demonstrated differential methylation between EOPE and spontaneous preterm birth, with p-values ranging from 3.80 E-5 to 0.036. Additionally, significant differences were noted at four CpGs between EOPE and fetal growth restriction, and 13 CpGs between EOPE and uncomplicated controls. The findings underscore a distinct DNA methylation profile in the placental tissues and UC-WBC of patients with EOPE, compared to controls, but no significant differences were found in patients with LOPE [16].

Regarding the pregnancy trimesters, DNA methylation data were analyzed for DMRs using the DMRFF method through three trimesters in a study. Within the first trimester, three DMRs were identified on chromosomes 5, 10, and 15. The most prominent region mapped to the RAB18 gene on chromosome 10 and included five CpGs. On the second trimester, DMRs were found on chromosomes 2, 10, and 19. The most significant region involved eight probes at the NCOA4 gene on chromosome 10. Lastly, on third trimester, DMRs were detected on chromosomes 6, 14, and 17, and the most significant DMR was linked to the TRAF3IP2-AS1/TRAF3IP genes on chromosome 6 [14].

Furthermore, a systematic analysis of DNA methylation patterns in placental tissues from women with EOPE and controls was undertaken, measuring approximately 27,000 CpG sites using an oligonucleotide microarray. The results demonstrated that PE is accompanied by specific changes in DNA methylation, particularly in EOPE where significant hypomethylation was observed in CpG loci that did not correlate with mRNA expression changes, suggesting distinct regulatory mechanisms at play [25].

Lastly, a comprehensive assessment of 1,505 CpG sites related to 807 genes in placental tissues from cases of EOPE revealed a substantial dysregulation, with 34 hypomethylated loci correlating with adverse pregnancy outcomes. The methylation aberrations of TIMP3 which were detected by other studies (See Sect."Gene-specific methylation patterns in placenta"), was also proposed as a prenatal diagnostic marker for this severe condition [31].

These studies collectively enhance our understanding of the molecular and epigenetic underpinnings associated with PE, underscoring the importance of epigenetic programming in placental and fetal health. ​The implications of these findings are significant, suggesting that DNA methylation may serve as a critical biomarker for the early identification and management of PE, potentially improving outcomes for both mothers and their infants.​ Further large-scale investigations focusing on the molecular pathways involved in these alterations are warranted to better elucidate their contributions to the pathophysiology of PE and its associated complications.

### Therapeutic and diagnostic implications

#### Methylation in placental tissue as a biomarker for early detection

A growing body of literature reports molecular and histological changes in the human placenta associated with PE. Placental DNA methylation and transcriptomic patterns have revealed molecular subgroups of PE linked to placental histopathology and clinical phenotypes. However, the clinical and molecular heterogeneity of PE complicates its study. To address this, researchers developed eoPred, a predictor of the DNA methylation signature associated with EOPE. Using public data from 83 placental samples (42 EOPE and 41 normotensive preterm births), they developed a supervised model based on a discriminative 45 CpG DNA methylation signature. eoPred's performance was assessed with cross-validation (AUC = 0.95) and an independent validation cohort (AUC = 0.725) [56]. A subset of fetal growth restriction and LOPE cases showed a similar DNA methylation profile, indicating overlapping placental pathology. eoPred was associated with gestational age and not driven by cell composition differences. This classifier should aid in studying placental insufficiency, facilitate identifying the EOPE placental phenotype, and standardize EOPE diagnosis for better cross-cohort comparisons. Lastly, eoPred will help investigate the relationship between placental pathology and genetic or environmental variables [56].

#### Non-Invasive DNA methylation biomarkers: cell-free DNA in maternal blood

Beyond tissue biomarkers, there is robust interest in translating methylation signatures into non-invasive diagnostic approaches by analyzing cell-free DNA (cfDNA) isolated from maternal plasma [57]. Recent work demonstrates that cfDNA methylation profiling, typically performed via reduced representation bisulfite sequencing (RRBS), can identify genome-wide methylation alterations that distinguish PE from uncomplicated pregnancies [58]. Machine learning models trained on top DMRs from cfDNA yield high specificity and AUC values (approximately 0.83–0.87) for symptomatic cases, while offering moderate sensitivity for predicting PE risk in early gestation [59]. These non-invasive strategies have clear clinical appeal, enabling early risk stratification and monitoring without the need for invasive placental sampling. Similar studies allowed for presymptomatic prediction as early as 12 weeks’ gestation (range 9–14 weeks), effectively within the first trimester, coming up with a model that could correctly predicted 72% of EOPE patients at 80% specificity [60].

The cfDNA methylation approach also offers logistical advantages: it is more automatable and cost-effective than other molecular platforms, and it allows for longitudinal sampling to observe dynamic changes throughout pregnancy [61]. Nevertheless, methodological hurdles remain—including the need for larger validation cohorts, precise accounting for gestational age and maternal covariates, and integration with established clinical and biochemical risk factors to improve diagnostic performance.

#### Integration of DNA methylation with other molecular ‘Omics’ data

Recent advances in multi-omics integration have further refined the diagnostic and prognostic utility of DNA methylation biomarkers for PE. Multi-layered models that combine DNA methylation with transcriptomic and proteomic data have demonstrated improved classification accuracy and sensitivity for various PE subtypes compared to single-omics approaches [38]. For example, machine learning systems leveraging integrated multi-omics data have achieved AUC values up to 0.94, with the identification of biological pathways such as tryptophan metabolism and oxidative stress signaling implicated in PE pathogenesis [38]. The amalgamation of methylation profiles with gene expression and protein markers enables a more comprehensive molecular understanding and facilitates the discovery of robust, reproducible biomarkers across diverse populations [62].

Furthermore, integrating DNA methylation data with established biomarkers (e.g., sFlt-1/PlGF ratio) enhances prediction models and could enable risk-adapted therapeutic decision-making [57]. However, clinical application requires harmonization of sample collection protocols, bioinformatic pipelines, and cohort phenotyping, in addition to large-scale validation.

#### Emerging therapeutic opportunities targeting DNA methylation

In parallel with diagnostic innovation, research is emerging on therapeutic strategies that target aberrant DNA methylation or related epigenetic modifications in PE. For example, low-dose acetylsalicylic acid (aspirin), already recommended for the prevention of PE in high-risk pregnancies, may exert beneficial effects in part by modulating placental DNA methylation and other epigenetic pathways, restoring cellular functions altered by disease processes [63]. Experimental models have also shown that DNA methylation inhibitors, such as 5-aza-2'-deoxycytidine, can reverse epigenetic silencing of key genes involved in trophoblast differentiation and placental function, providing proof-of-principle for potential epigenetic therapies [64]. However, their translation to the clinic remains challenged by concerns regarding specificity, safety, and off-target effects in the maternal–fetal context.

**Fig. 3:**
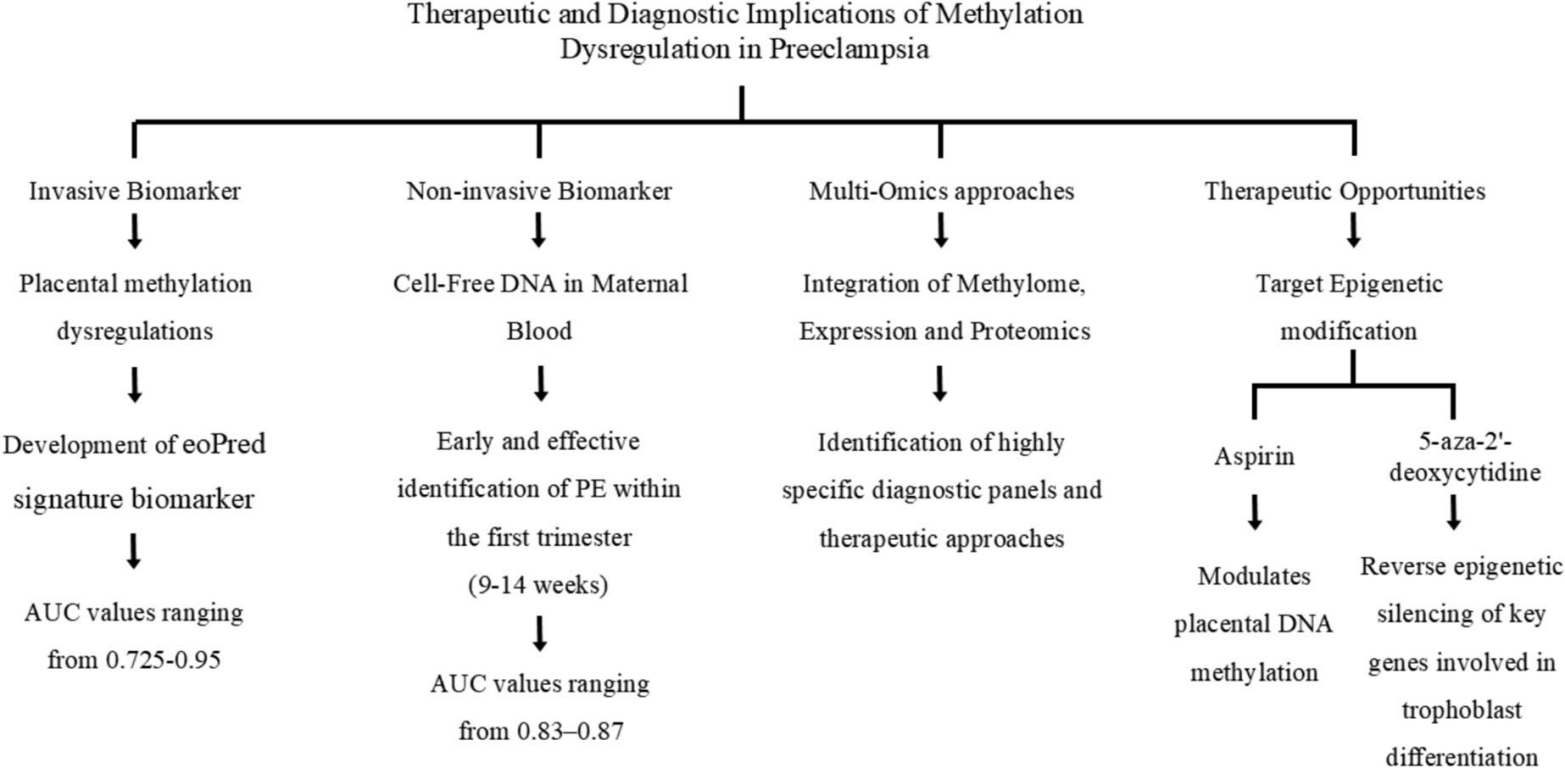
Overview of diagnostic and therapeutic implications of placental methylation in preeclamptic patients

Central to therapeutic translation is a robust mechanistic understanding: evidence suggests that oxidative stress, a well-documented feature of PE, interacts with DNA methylation machinery to drive gene expression dysregulation, thereby presenting rational points for intervention. Multi-omic analyses further aid in prioritizing therapeutic targets by revealing upstream regulators and affected networks shared among PE subtypes (Fig. 3).

## Discussion

**Fig. 4:**
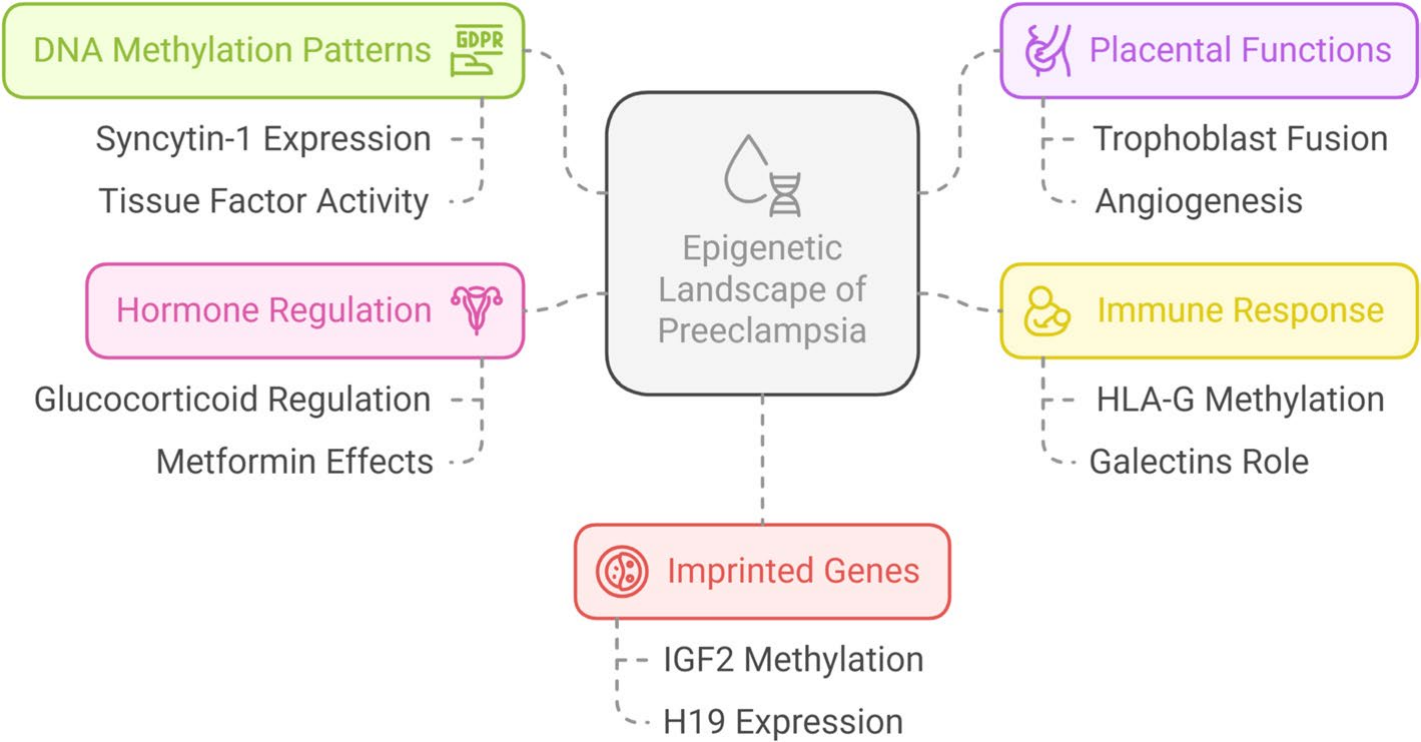
This schematic diagram summarizes the current understanding of epigenetic dysregulation in preeclampsia. It integrates multiple layers of epigenetic alterations—including aberrant DNA methylation, imbalanced imprinted gene expression (e.g., IGF2, H19), and disrupted hormone signaling pathways—that collectively influence placental development, vascular remodeling, and immune modulation. The figure further outlines how these modifications converge with genetic and environmental factors to contribute to the pathogenesis of preeclampsia, offering a unifying framework for future research and potential therapeutic strategies

The discussion of our findings reveals several key insights into the epigenetic landscape of PE. Our systematic review demonstrates that DNA methylation plays a crucial role in PE pathogenesis, with distinct patterns emerging between EOPE and LOPE. The comprehensive analysis of genome-wide methylation studies has revealed significant differential methylation patterns in placental tissue, particularly in genes involved in trophoblast function, angiogenesis, and immune response (Fig. 4).

Recent evidence suggests that abnormal DNA methylation in placental tissue is associated with various aspects of PE pathophysiology [65]. For instance, studies have shown that syncytin-1, which is mainly expressed by trophoblast cells and mediates trophoblast fusion, exhibits decreased expression in PE placentas due to promoter hypermethylation [47, 66]. This finding highlights the importance of DNA methylation in regulating key placental functions [67]. Furthermore, research has demonstrated that overexpression of methyltransferases (e.g. DNMT3a) could be responsible for decreased syncytin-1 expression through promoter hypermethylation, which subsequently inhibits trophoblast cell fusion [47].

The role of immune-related genes in PE development has emerged as a significant area of investigation [68]. Tissue factor (TF), a membrane glycoprotein crucial for fetal and placental development, shows increased expression in PE placentas accompanied by significantly reduced DNA methylation of its promoter [69]. This altered expression may contribute to the loss of immunosuppressive function and hinder the development of placental immunological tolerance [70]. Additionally, galectins, which are highly expressed at the maternal–fetal interface, show distinct methylation patterns that affect immune tolerance, with three genes in a Chr19 cluster primarily conferring immune tolerance in maternal–fetal interactions [71].

Human leukocyte antigens (HLA) molecules have emerged as critical players in maternal–fetal immune tolerance [72]. Research has shown that HLA-G, which is abundantly expressed on extravillous trophoblasts, exhibits extremely high levels of CpG methylation in its promoter region in PE placentas. This finding is particularly significant as HLA-G promotes fetal-maternal immune tolerance by reducing NK cell toxicity and cytokine production. The study demonstrated that DNMT1-mediated promoter hypermethylation of HLA-G is associated with PE development [45].

The relationship between DNA methylation and placental hormone regulation presents another critical aspect of PE pathophysiology [43, 73]. Glucocorticoid regulation, particularly through the enzyme 11β-hydroxysteroid dehydrogenase 2 (11βHSD2), plays a vital role in protecting the fetus from excessive exposure to maternal glucocorticoids [33]. Studies have shown that EOPE is associated with increased DNA methylation levels at CpG sites of the NR3C1 and HSP90AA1 genes [74], which is responsible for cortisol effects. This finding suggests a potential mechanism by which epigenetic modifications might influence hormone regulation in PE.

Recent investigations have also revealed the importance of imprinted genes in PE development. For instance, IGF2, which is maternally imprinted and paternally expressed, shows altered DNA methylation patterns in EOPE placentas. These changes affect not only placental tissue but also umbilical cord leukocytes and vein endothelial cells, suggesting widespread epigenetic dysregulation in PE [21]. The therapeutic implications of these findings are particularly noteworthy. Recent clinical trials examining the effectiveness of metformin in PE treatment have shown promising results, with the drug affecting H19 gene DNA methylation and expression. The therapeutic benefit of metformin appears to be associated with a dose-dependent increase in H19 methylation and suppression of H19 expression, suggesting potential therapeutic strategies targeting specific methylation patterns [32].

However, it is crucial to acknowledge certain limitations in current research. The study of placental DNA methylation patterns is complicated by the heterogeneity of PE presentations and the influence of various factors such as gestational age and fetal gender. A significant challenge arises from gestational age discrepancies, particularly in studies comparing EOPE with spontaneous preterm controls delivered around 32 weeks, since preterm birth itself impacts DNA methylation and thus acts as a major confounder, making it difficult to distinguish methylation alterations attributable specifically to PE from those related to prematurity [65]. Furthermore, studies examining LOPE (34–36 weeks) often rely on growth-normal preterm controls and frequently report weak or minimal methylation signal differences, suggesting either a subtler epigenetic signature in this subgroup or a greater degree of biological heterogeneity, which further complicates the detection of robust biomarkers [75]. Additionally, the relationship between DNA methylation and gene expression is not always straightforward, as some studies demonstrate discordant patterns between methylation status and gene expression levels [76].

Importantly, the interpretation of placental DNA methylation data must consider the tissue’s inherent cellular heterogeneity. The placenta comprises a complex mix of cell types—such as cytotrophoblasts, syncytiotrophoblasts, stromal cells, endothelial cells, Hofbauer cells, and various immune cells—each with distinct methylation profiles [77]. Studies have demonstrated that variations in placental cellular composition strongly impact DNA methylation analyses; for example, differential proportions of trophoblast subtypes or immune cells can confound bulk tissue methylation patterns and obscure true disease-associated epigenetic changes [78]. Adjusting for cell type composition using reference-based deconvolution methods significantly improves the accuracy of methylation analyses and helps distinguish true epigenetic alterations related to PE from those arising due to cell type differences [78]. In addition, recent analyses revealed that cellular heterogeneity is not only a confounder but can itself be a reflection or driver of disease, as PE-associated changes in cell type proportions (such as an increase in trophoblast lineages and decrease in stromal or Hofbauer cells) can directly impact observed methylation patterns [79]. Therefore, future studies should incorporate computational estimation and adjustment for cell type heterogeneity or, where possible, analysis of purified cell populations from placenta to provide clearer insights into the epigenetic mechanisms underlying PE.

These findings collectively demonstrate the complex interplay between DNA methylation and PE pathogenesis, highlighting the potential for epigenetic-based therapeutic interventions. The identification of specific methylation patterns associated with different aspects of PE development provides valuable insights for future research directions and potential therapeutic strategies.

## Future directions and research gaps

Our systematic review has identified several critical gaps in the current understanding of placental epigenetics in PE.​ First, current methylation analysis methods cover only approximately 2% of CpG sites in the human genome, potentially missing significant epigenetic modifications. Additionally, the relationship between DNA methylation and gene expression requires further investigation, as evidence suggests a complex regulatory mechanism beyond simple methylation patterns. The role of tissue-specific methylation patterns within different placental cell types remains unclear. While studies have identified distinct methylation profiles, the functional implications of these differences and their contribution to PE pathogenesis need further exploration. Moreover, the temporal dynamics of methylation changes throughout pregnancy require more comprehensive investigation, as most studies focus on term placentas or specific time points. Lastly, there is a significant need for studies investigating the potential therapeutic applications of these epigenetic insights. While current research has identified numerous differential methylation patterns, the translation of these findings into clinical interventions remains a crucial gap. Future studies should focus on developing integrated approaches that combine multiple epigenetic markers and validation of potential therapeutic targets based on these methylation patterns.

## Conclusion

This systematic review provides comprehensive insights into the epigenetic landscape of placental tissue in PE. The identified methylation patterns not only enhance our understanding of PE pathophysiology but also offer potential biomarkers for early detection and therapeutic targets. The distinct methylation profiles between EOPE and LOPE suggest different pathogenic mechanisms, highlighting the need for tailored therapeutic approaches. While challenges remain in translating these findings into clinical practice, our review establishes a strong foundation for future research in this critical area. ​The significance of these epigenetic modifications in PE development underscores their potential as therapeutic targets and diagnostic tools, ultimately contributing to improved maternal and fetal outcomes​.

## Data Availability

No datasets were generated or analysed during the current study.
